# Supplementary material for: Extending Field Limits in Nanoscale Magnetic Imaging With Metamaterial‐Inspired Magnetic Flux Concentrators
Source: Small. 2026 Jun 30;22(43):e00073. doi: 10.1002/smll.202600073 (PMC13432482; doi:10.1002/smll.202600073)
Supplement: Supplementary file 1 — Supporting File 1: smll74031‐sup‐0001‐SuppMat.docx. [file SMLL-22-e00073-s001.docx]

**Supporting information**

**Extending Field Limits in Nanoscale Magnetic Imaging with Metamaterial-inspired Magnetic Flux Concentrators**

A. Barrera^1,ǂ^, E. Fourneau^2,ǂ^, T. Pirottin^2,3^, L. Marcano^4^, R. Abrudan^5^, R. Huang^6^, Ll. Balcells^1^, I. Orue^7^, M.L. Fdez-Gubieda^8^, D. Villanueva^8^, A.G. Gubieda^9^, L. Chang^6^, B. Vanderheyden^3^, R.J. Harrison^10^, A.V. Silhanek^2,*^, A. Palau^1,*^ and S. Valencia^5,*^

^1^Institut de Ciència de Materials de Barcelona, ICMAB-CSIC, Campus de la UAB, 08193 Bellaterra, Spain

^2^Experimental Physics of Nanostructured Materials, Q-MAT, Department of Physics, Université de Liège, Sart-Tilman B-4000, Belgium

^3^Montefiore Research Unit, Department of Electrical Engineering and Computer Science,

Université de Liège, B-4000 Sart Tilman, Belgium

^4^Departamento de Física, Universidad de Oviedo, Calvo Sotelo s/n, 33007, Oviedo, Spain

^5^Helmholtz-Zentrum Berlin fur Materialien und Energie GmbH, Berlin, Germany

^6^Peking University, Beijing, China

^7^SGIker, Universidad del País Vasco – UPV/EHU, 48940 Leioa, Spain

^8^Dpto. Electricidad y Electrónica, Universidad del País Vasco, 48940 Leioa, Spain

^9^Dpto. Inmunología, Microbiología y Parasitología, Universidad del País Vasco – UPV/EHU, 48940 Leioa, Spain

^10^Department of Earth Sciences, University of Cambridge, U.K.

**1.-** **Effective field determination of MFCs based on XMCD spatially resolved imaging.**

Micromagnetic simulations were performed to compute the intensity and spatial distribution of the concentrated field within the gap. A flower-like MFC with 10 petals, as described in panel (a) of Figure S1 in the main manuscript, was modeled using the following geometrical parameters: an outer radius $R_{o}=10$µm, an inner radius $R_{i}=2$µm, a thickness $t=70$ nm, and a gap width $d_{gap}=500$ nm. The material parameters used in the simulation are reported in the Methodology section of the main manuscript, along with the rationale for the device downscaling relative to the experimental structure. The results shown in Figure S1a present the variation of the concentrated field $\mu_{0}H_{con}$ as a function of the external field, evaluated at different positions inside the gap, equidistant from both edges. Step-like magnetization jumps observed in the micromagnetic simulations arise due to due to abrupt changes of magnetic domain arrangement such as the formation of flux -closure states. A more gradual magnetization reversal is expected for larger structures as they allow a larger number of domains and a more gradual and spatially averaged magnetization reversal.

The data demonstrate that the concentrated field remains relatively homogeneous across the central region of the gap (for $y$ between −1 µm and +1 µm). Minor variations are attributed to the inhomogeneity of the domain distribution.


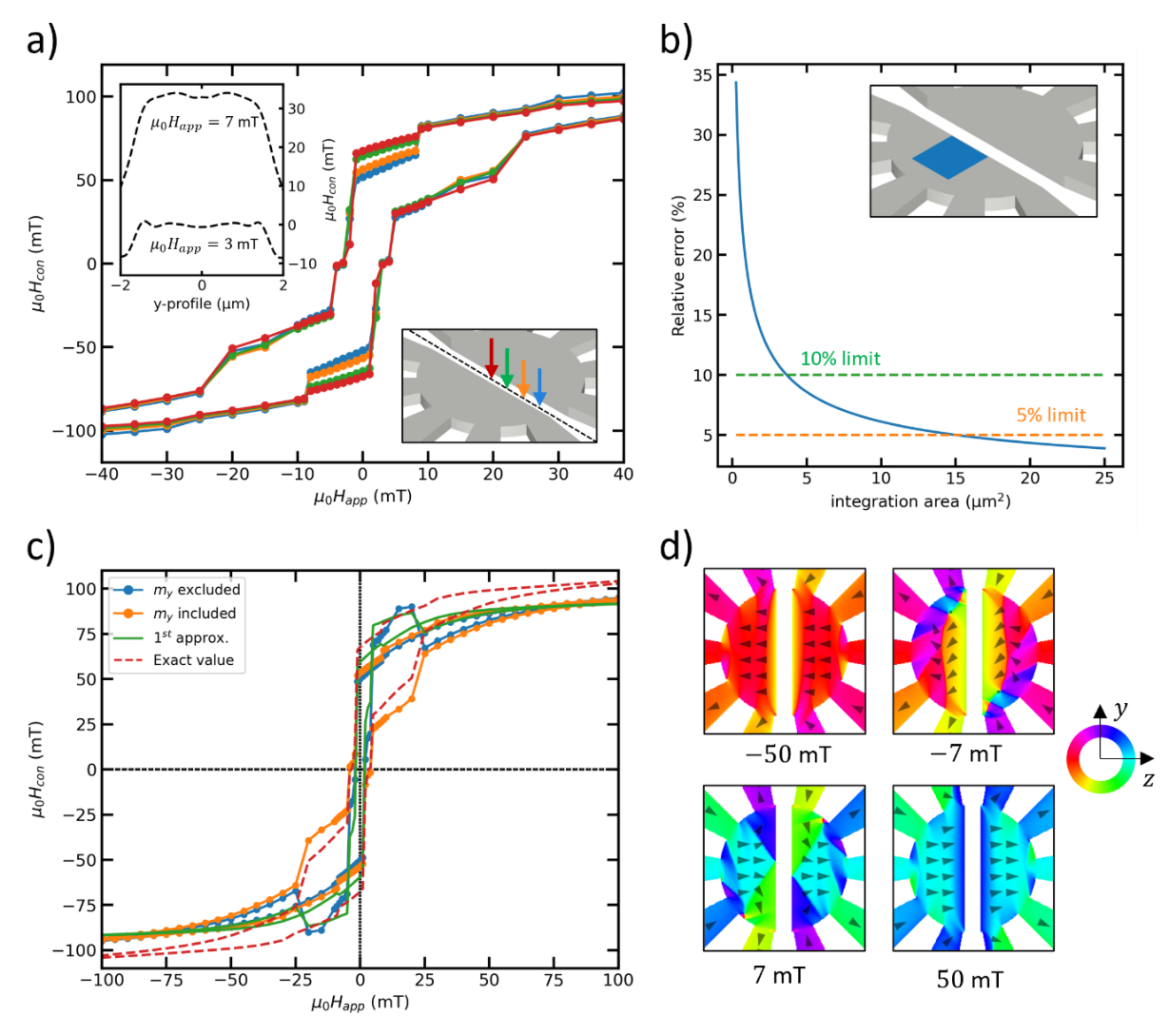


**Figure S1** Micromagnetic simulations. (a) Variation of the concentration field probed at different locations inside the MFC gap (illustrated in the lower-right inset) as a function of the applied field. The top-left inset shows the change of $H_{con}$ along the y-axis inside the gap. (b) Variation of the relative error for the calculation of $H_{con}$ depending on the area of the top and bottom surfaces of the integration volume. (c) Variation of the concentration field probed at the center of the MFC gap as a function of the applied field using different approximations. (d) Colored maps of the magnetic moment distribution in the MFC for four different applied fields.

The field generated in the gap corresponds to the collective contribution of all magnetic moments composing the MFC and can be computed using the dipolar stray field:
$\mu_{0}H_{z}=\int_{V} \frac{\mu_{0}}{4\pi}\left[ 3\frac{z\left( m_{z}z+m_{y}y \right)}{r^{5}}-\frac{m_{z}}{r^{3}} \right]dV$,
with $r=\sqrt{z^{2}+y^{2}}$ representing the distance from the dipole and $m=m_{x}\hat{x}+m_{y}\hat{y}+m_{z}\hat{z}$ is the magnetic moment. Since the dipolar field decreases with $r^{-3}$, the magnetic field in the gap is primarily determined by the magnetic material located in the vicinity of the probed position. The minimum integration volume required to accurately estimate the concentrated field can be evaluated by considering a saturated sample ($m_{z}=1$) with infinite dimensions (i.e., $R_{o}>R_{i}\gg d_{gap}$). Figure S1b shows the relative error on $\mu_{0}H_{con}$ as a function of the integration area, defined as a square with one of its edges in contact with the gap edge. The results indicate that the error decreases to less than 5% when considering a square integration area of $4\times4$ µm². It should be noted that such a region is small compared to the inner core of the MFCs fabricated for this work but much larger than the one used in the simulations.

The same method of integrating over a restricted area was used to compute the concentrated field inside the gap.

The calculation was performed first by considering only the $m_{z}$component and then including both $m_{z}$and $m_{y}$. The resulting hysteresis loops are reported in Figure S1c and compared with the reference $\mu_{0}H_{con}$ (integrated over the entire sample, shown as a dashed line) and with a first-order approximation $\mu_{0}H_{con}=\alpha m_{z}$ (solid line), assuming that the magnetization reversal behaves as a Stoner–Wohlfarth particle (i.e., a single-domain system undergoing coherent rotation under variation of the external field). The results show that:

1. The concentrated field is qualitatively captured by the local integration of both $m_{z}$ and $m_{y}$, as the corresponding hysteresis loop closely resembles the reference one. The quantitative discrepancy is attributed to the limited size of the integration volume.
2. The results obtained by neglecting the $m_{y}$ component are close to those including both $m_{z}$ and $m_{y}$, except for applied fields between 5 and 25 mT. In this range, $\mu_{0}H_{con}$ increases only slightly with the external field, compared to the marked increase observed in the 0–5 mT range, even though saturation is not yet achieved. This behavior is due to the formation of a flux-closure pattern in the core of the device, as illustrated in panel (d), which shows color maps of the magnetization distribution for different applied fields. In such situations, the concentrated field is mainly generated by volume magnetic charges (inside domain walls) rather than by surface charges along the gap edges. Therefore, in this configuration, accurate computation of the concentrated field within the gap requires knowledge of the transverse component of the magnetization, whereas $\mu_{0}H_{con}$can otherwise be estimated solely from the component aligned with the external field.
3. The first-order approximation accurately predicts the switching field but shows limited quantitative accuracy for the magnitude of the concentrated field.

The importance of the $m_{y}$ component in the calculation of $\mu_{0}H_{con}$ is clearly demonstrated for small structures such as the one simulated. However, for experimental structures that are ten times larger, the magnetic domains are expected to be significantly larger than the minimal integration area required to accurately compute $\mu_{0}H_{con}$. Consequently, in the absence of domain walls within the integration area near the sensing point in the gap, the z-component of the concentrated field can be derived from the z-component of the magnetization alone. Interestingly, this hypothesis can be verified by comparing the shape of the hysteresis loops of the integrated region of the MFC and of the sample under investigation (e.g., the magnetosome), both obtained from XPEEM measurements (Figures 2 and 3 of main manuscript). The absence of discrepancies such as those observed between $\mu_{0}H_{con}$ and $m_{z}$ in the simulated device supports the hypothesis that the concentrated field can indeed be computed from the z-component of the MFC magnetization alone.

On this basis, we computed the field *μ_0_H_con_* created by the MFCs used in the present study from the spatial distribution of the XMCD signal - which is proportional to the z-component of the MFC magnetization - as function of the applied field. Figures 2e and 3e of the main manuscript show the regions XMCD signal used. For the fossil, the XMCD signal corresponding to the left-hand side of the MFC looks noisier than that corresponding to the right-hand side. This is due to the presence of foreign magnetic material collected during the magnetic extraction of the fossil (Figure S2). This material is covered by Co during the fabrication process of the MFC. The XMCD signal in shadowed regions (dark areas in XAS images) reflects both, the magnetic properties of the Cobalt layer and magnetic contributions of the material in the beam path^1^. To overcome this limitation, and for that particular case, the right-hand side XMCD distribution has been mirrored in order to compute *μ_0_H_con_*.


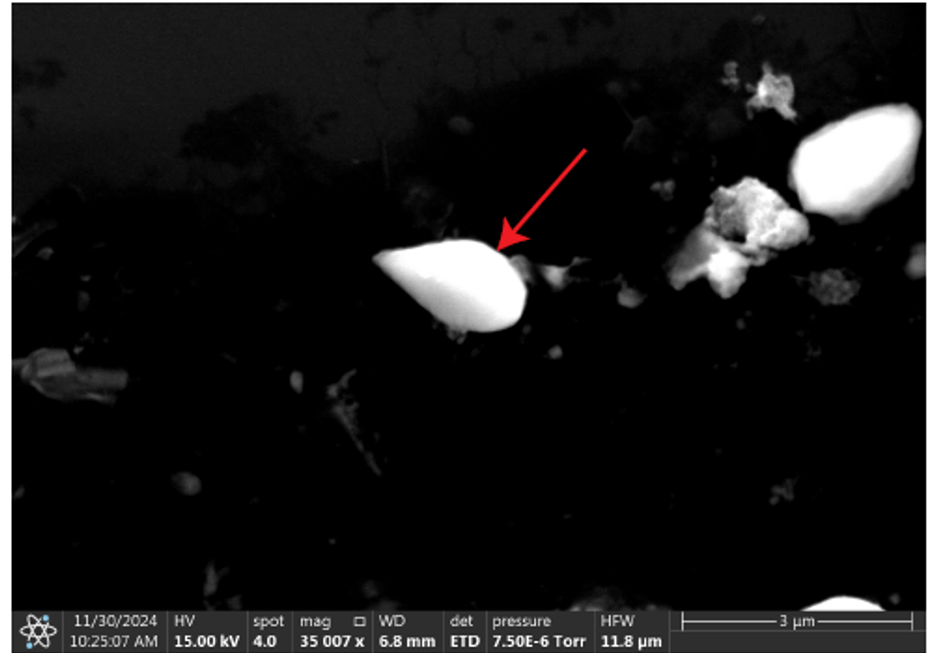


**Figure S2** Scanning electron microscopy image of the fossil and surrounding area showing the presence of additional magnetic material collected during the magnetic extraction of the fossil Red arrow points to the fossil, at its right we see additional material collected during the magnetic extraction process.


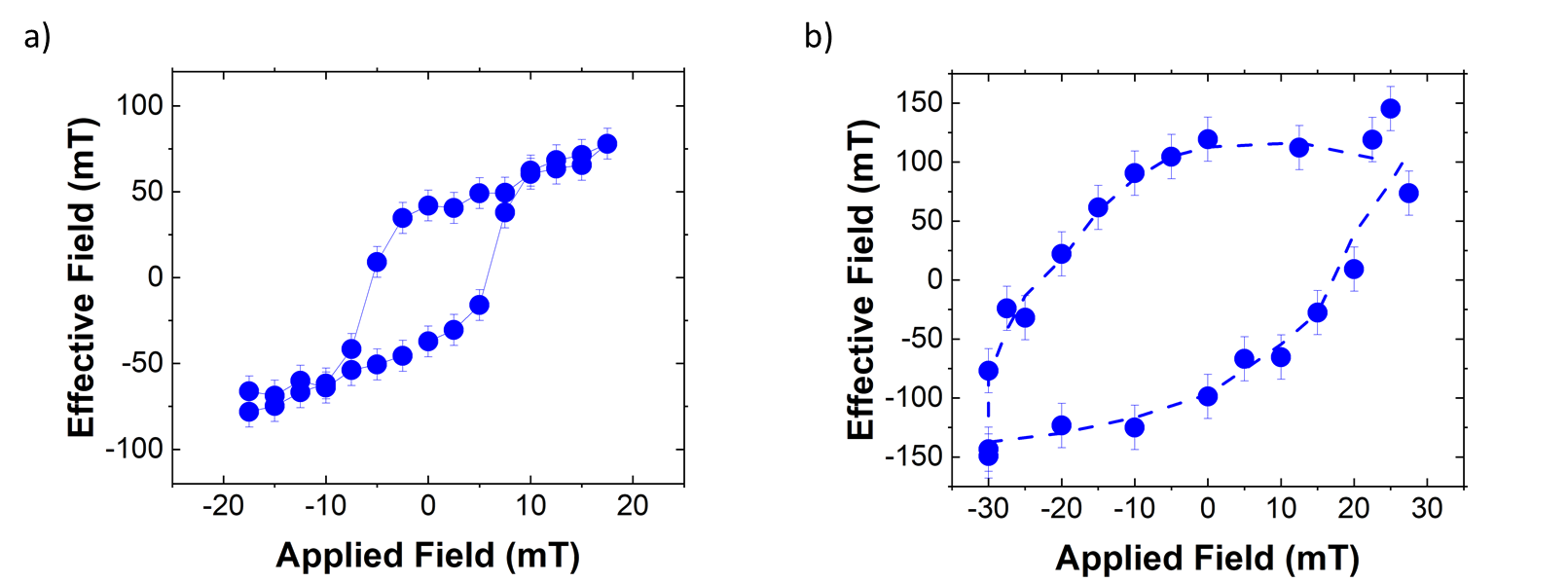


**Figure S3** Effective field reached with the use of the MFCs as function of the applied field within the PEEM for the MFCs employed for the a) magnetosomes chain and b) giant magnetofossil. Lines connecting points are shown as guides to the eye. The effective fields have been computed by considering the space-resolved XMCD data which is proportional to the magnetization component of the MFC across the gap direction. Error for the effective field determination of the MFC corresponding to the magnetosome chain is ± 9 mT. For the MFC used for the fossil the error is ±19 mT. See Supporting Information section 1.1.

Panels a and b from Figure S3 show the effective field -defined as the sum of the field created by the concentrator and the applied field- as function of the latter, at the magnetosome chain and fossil position, respectively. In both cases, fields exceeding 100 mT can be achieved with applied magnetic fields of approximately 30 mT.

- 1. **Intrinsic error in the determination of the effective Vs applied field curve in PEEM.**

The method proposed here to determine the effective field (*H_eff_* = *H_app_* + *H_con_*) created by the MFC at its gap center is based on the measurement of the XMCD asymmetry to compute *of μ_0_H_con_*. To determine the effective field error (ε_eff_) we adopt the following assumptions: i) H_app_ is considered to be error free (ε_app_=0) so that ε_eff_ is equal to the error in the determination of μ_0_H_con_ (ε_con_), and ii) we consider a uncertainty in the XMCD asymmetry determination of ±0.005. This error represents approximately 12.5% of the XMCD signal measured at saturation for the MFCs employed for both the fossil (XMCD = 0.04) and 4% for the MFC for the magnetosome chain (XMCD = 0.12). This translates to an error ε_con_ equal to 12.5% and 4% of μ_0_*H*_con_ at saturation (~ 110 mT and 80 mT, respectively) which corresponds to ε_con_ = ±14 mT and ε_con_ = ±5 mT for fossil and chain.

The error in the determination of the effective field for the MFC used for the fossil is larger than that used for the magnetosome chain due to the low XMCD signal which decreases the signal to noise ratio.

The MFC used for the fossil sample presents an additional source of uncertainty arising from the presence of foreign magnetic material in the vicinity of the fossil. This material is present due to the magnetic extraction procedure followed to obtain the sample (Figure S2). In the worst-case scenario, i.e. assuming full saturation over the entire loop, particles of magnetite (Ms ≈ 4.8 × 10^5 A/m) with effective diameter equal to its large 2D axis and a collinear alignment, and taking into account measured distance from the fossil center, the accumulated stray field contribution of these particles at the fossil position can be shown to be ≲5 mT. In this case ε_eff_ = ε_con_ ± 5mT, so that ε_eff_ = ±19 mT.

Similarly, for the magnetosome chain, the presence of additional magnetosomes close to the main chain, assuming a colinear alignment, introduces an accumulated stray field contribution of ±4 mT. In this case ε_eff_ = ±9 mT.

**2.- Micromagnetic simulations for the giant magnetossil.**

**2.1 Near-surface restricted simulated magnetic hysteresis loops.**

Magnetic hysteresis loops corresponding to the z-component of the fossil´s magnetization depicted in Figure 3i and 4j of the main manuscript have been obtained by averaging the full particle volume. When the average magnetization is restricted to a near-surface region of the fossil, the simulated hysteresis depends strongly on the azimuthal orientation as the three-dimensional vortex state implies a continuous family of distinct hysteresis loops, each associated with a different azimuthal orientation of the fossil. Consequently, a comparison between averaged surface-sensitive XMCD data and surface-restricted simulated curves becomes non-trivial due to this rotational degree of freedom, see Figure **S4** and Figure S5.


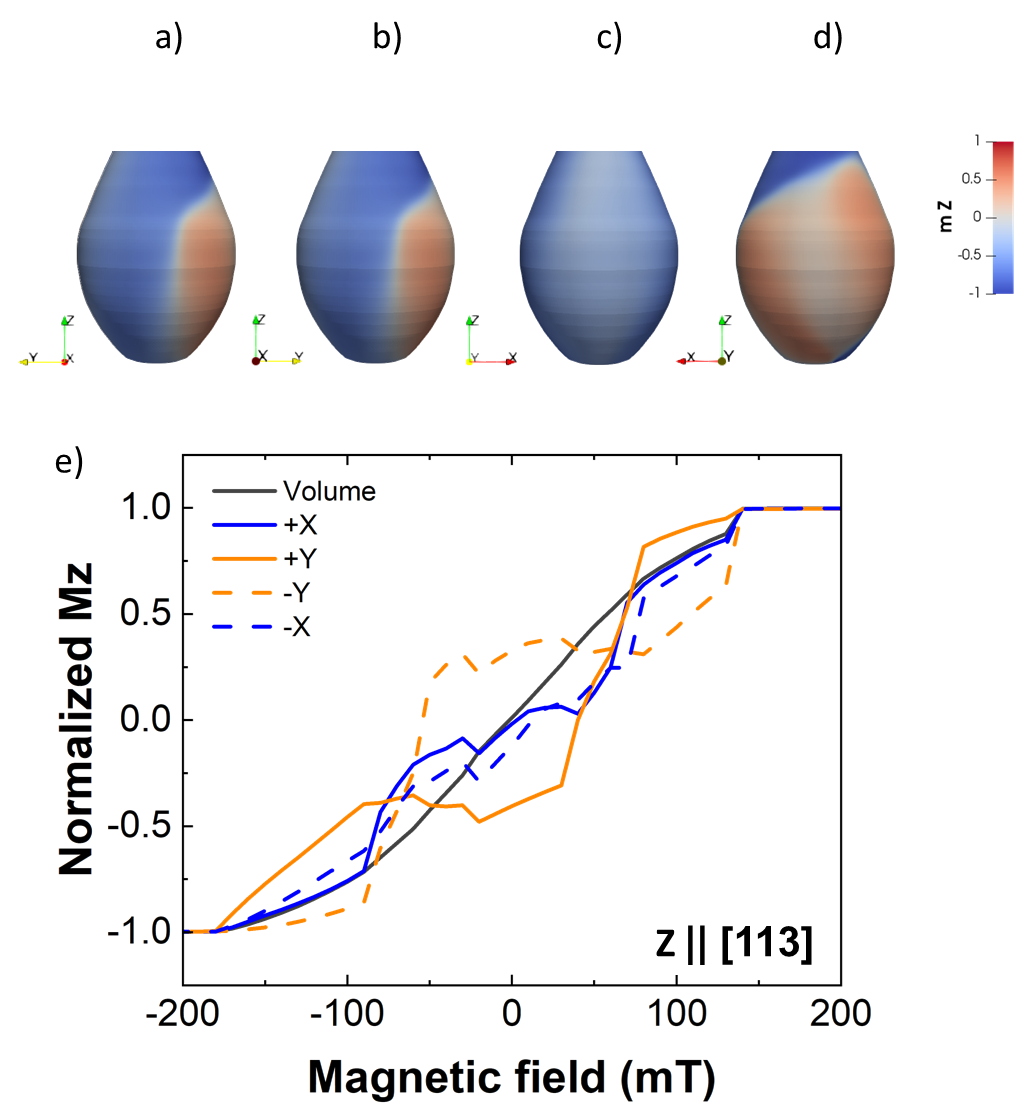


**Figure S4** Micromagnetic simulations. Space resolved z-component of the surface magnetization for the giant magnetosfossil for an applied field of 40 mT (after saturation at -300 mT as seen along the a) +X, b) -X, c) +Y, and d) -Y direction. The crystallographic orientation along the long axis of the particle has been set to [113]. e) Corresponding magnetic hysteresis loops of the Z-component of the magnetization. The volume-averaged data (black curve) is compared with near-surface averaged loops (5 nm depth) along *X, -X, +Y, and -Y directions. To mimic the PEEM experiment, the tip of the fossil has been excluded from the averaging.


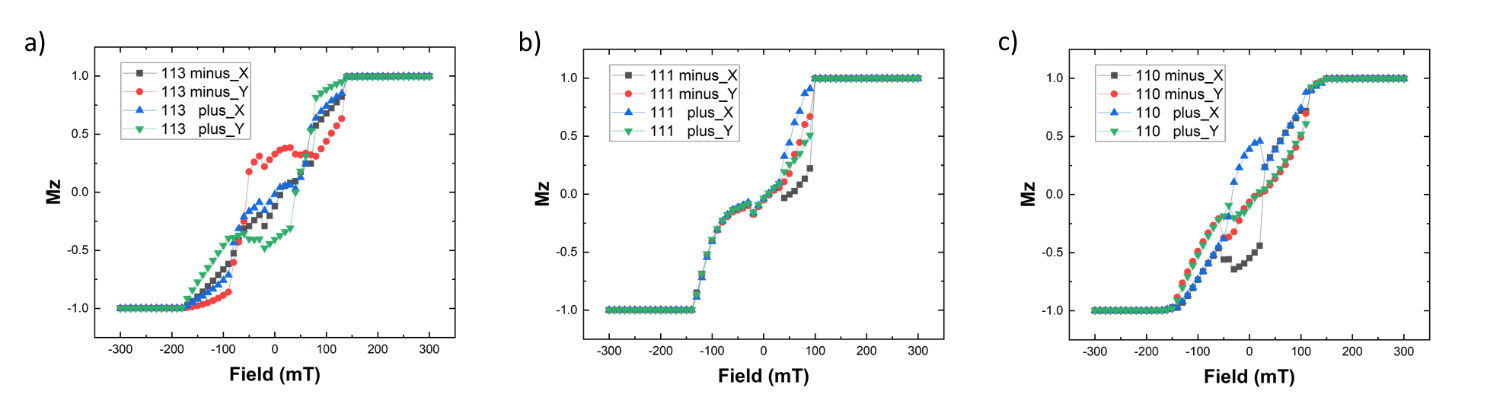


**Figure S5** Magnetic hysteresis loop obtained for the Giant Magnetofossil with near-surface (5 nm depth) magnetic hysteresis loops along +X, -X, +Y, and -Y directions obtained from micromagnetic simulations considering [113] (a), [111] (b), and [110] (c) crystallographic orientations along the long axis of the spearhead giant magnetofossil. To mimic the PEEM experiment, the tip of the fossil has been excluded from the averaging.

**2.2 Field dependence of the magnetic domain structure.**

Figure 5 within the main text depicts images of the surface magnetic domain structure of the giant magnetofossil as a function of the magnetic field viewed along the +X direction and obtained from micromagnetic simulations considering [113], [111], and [110] crystallographic orientations along the long axis of the spearhead. For completeness, Figure S6 shows the corresponding surface magnetic domain structure viewed along the +Y direction.


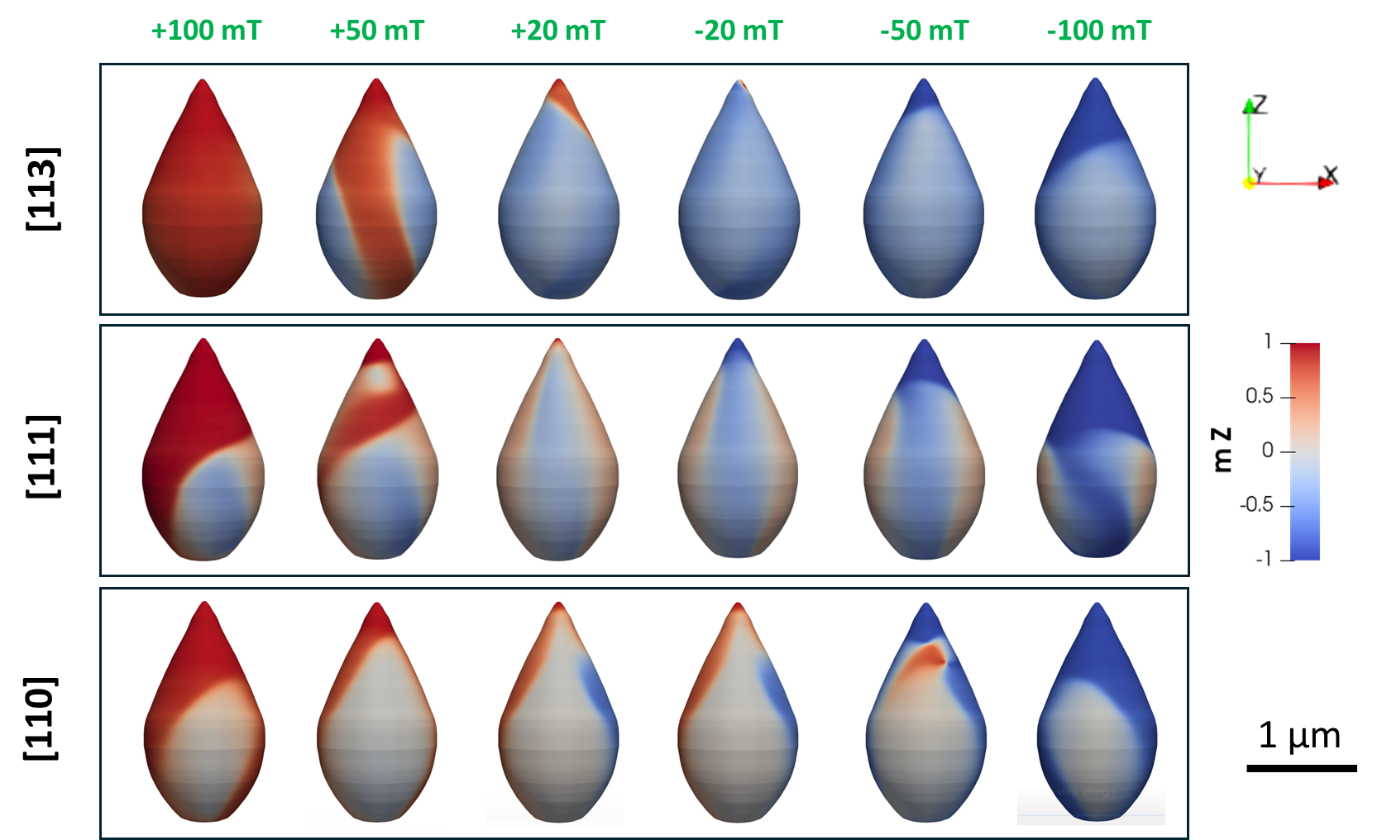


**Figure S6** Micromagnetic simulations of the imaged spearhead giant magnetofossil grain considering different crystallographic orientations. View along +Y direction of the z-component of the magnetization of the giant magnetofossil as function of a magnetic field applied along its long axis (z) after magnetic saturation at +300 mT. Top: [113] crystallographic orientation along z, middle: [111] crystallographic orientation along z, and bottom: [110] crystallographic orientation along z.

**2.3 Local influence of the Co covering the particle tip**

The deposition of the flux concentrator resulted in the tip of the magnetite magnetofossil becoming coated in Co (Figure 3b). To explore how magnetostatic interaction between this Co coating and the underlying magnetite might influence the hysteresis behavior, a two-phase micromagnetic model was created to approximate the Co-covered tip (Figure S8a-c). Each phase was meshed with a 20 nm resolution. Although this is far coarser than would normally be desired for Co, it is sufficient for our purpose here, which is simply to model the influence of the stray field of the magnetized Co coating, rather than model the specific details of its internal domain structure.

In the micromagnetic simulations, we neglect exchange coupling between the Co layer and the Fe₃O₄ fossil. This assumption is justified by the nature of the interface: the fossil originates from a natural sedimentary environment, and spatially resolved spectroscopic measurements (see Figure S7) reveal the presence of Ca at the surface, likely associated with residual mineral phases. Such interfacial layer is expected to disrupt direct metallic contact between the Co and magnetite, thereby suppressing exchange coupling.


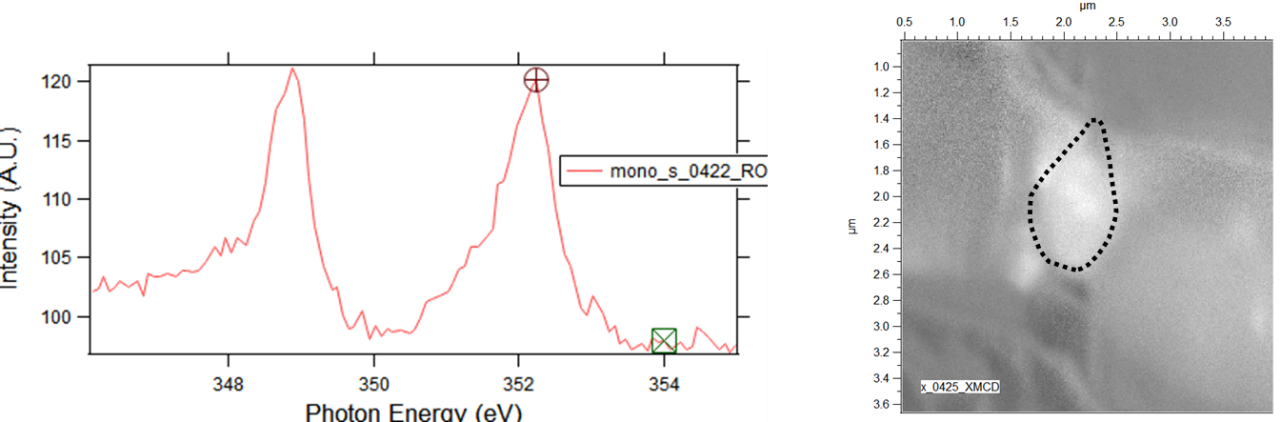


**Figure S7** Left) Spectrum across Ca M-edges obtained by averaging the signal over the fossil area delimited by the dotted line in right panel. Right) Image obtained by computing the ratio of images obtained at the Ca edge (round marker in right panel at ca. 352.2 eV) and at the pre-edge (square marker in right panel at 354.0 eV).

A comparison of the volume averaged hysteresis loop of the magnetite particle simulated with (red solid line) and without (black dashed line) the presence of Co is shown in Figure S8d. The main impact of the Co is to open the loop and to create a slight positive (negative) magnetization bias for positive (negative) applied fields. The magnetization bias can be seen clearly by comparing snapshots of simulations performed at +50 mT (Fig. S7e, f). The simulation with Co present (Figure S8f) shows an expanded positive (red) domain around the tip. This expanded domain survives at remanence (Figure S8h), whereas the model without Co relaxes to the expected single-vortex state (Figure S8g).

**
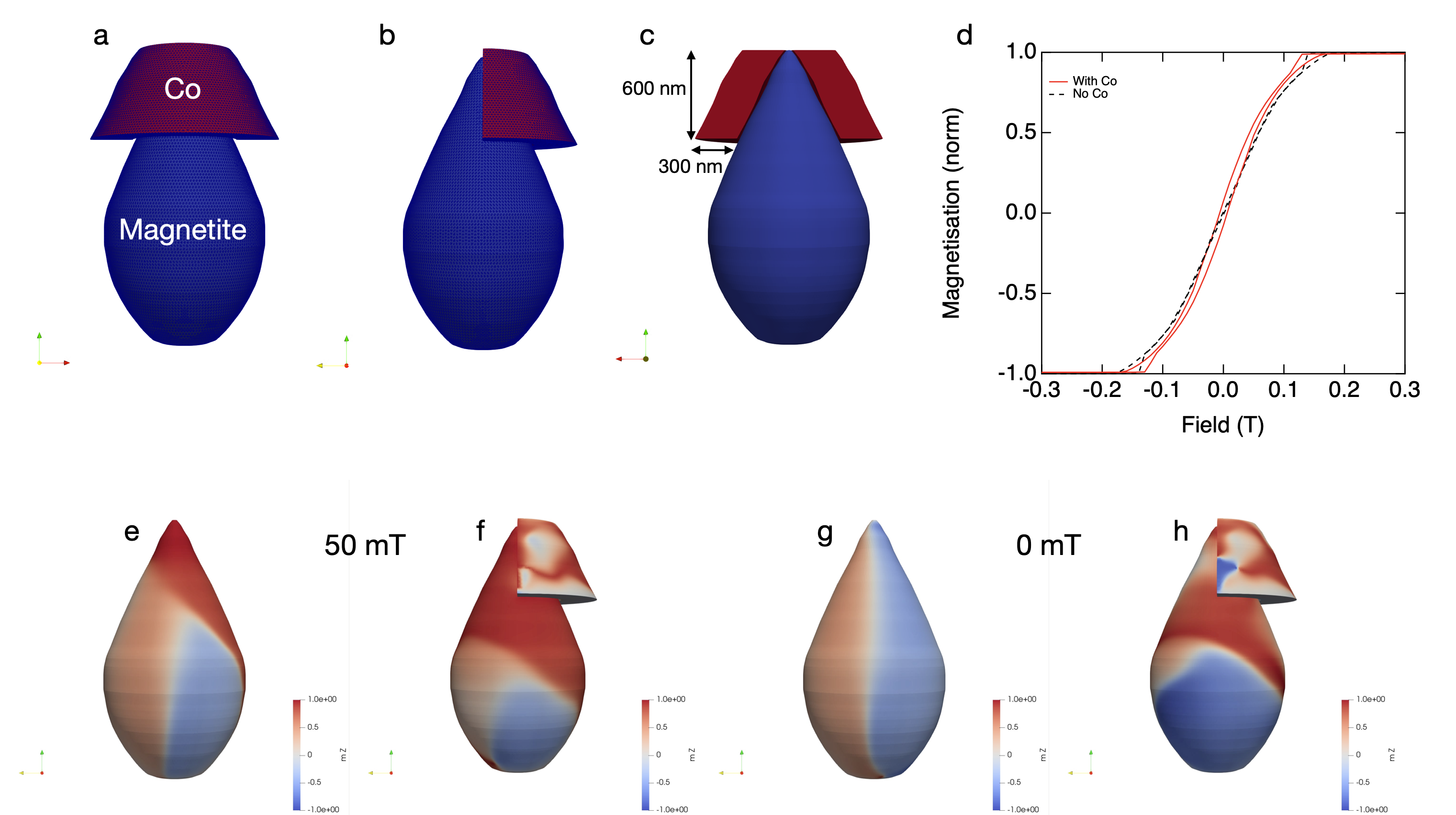
**

**Figure S8** Result of micromagnetic simulations considering the partial coverage of the tip of the fossil by 400 nm Co as observed experimentally. a)-c) model used for simulations. d) Comparison of volume integrated magnetic hysteresis loops for the fossil with (red) and without (black) cobalt coverage. e)-f) Image of the magnetic domain state at 50 mT as seen from +X direction without and with Co coverage. g)-h) Image of the magnetic domain state at 0 mT as seen from +X direction without and with Co coverage.

**2.4 Quantitative comparison between spatially resolved XMCD data and micromagnetic simulations**

Comparison of the experimentally observed magnetization reversal process of the fossil, measured by XMCD, with micromagnetic simulations for the [113], [111], and [110] crystallographic orientations along the long axis of the fossil allows us to exclude the [110] orientation. Simulated magnetization maps for the [110] orientation (bottom panels of Figures 5 and S6) display extended regions of the fossil where the z-component of the magnetization is nearly zero. Experimentally, this would appear as large areas with XMCD close to zero (whitish areas), which is not observed.

A quantitative comparison of the spatially resolved XMCD data with the magnetization maps obtained from micromagnetic simulations allow us to distinguish between the two remaining possibilities, namely [113] and [111] orientations. This can be achieved by comparing how the absolute value of the z-component of the magnetization |Mz| of the fossil evolves with the (effective) magnetic field. To this end, we integrate the spatially resolved absolute value of the XMCD signal, which is proportional to Mz, and compare it with the |Mz| values expected from micromagnetic simulations. For the latter and for the sake of comparison with the experiment, the integration has been restricted to a near-surface region of 5 nm and the tip of the fossil has been excluded. The results are shown in Figure S9. The experimentally observed decrease of |Mz| at low fields (blue dots) agrees with the expected behavior predicted for a [113] crystallographic orientation along the long axis of the fossil (panel a). For [111] (panel b) the decrease of |Mz| is larger than what is experimentally observed.


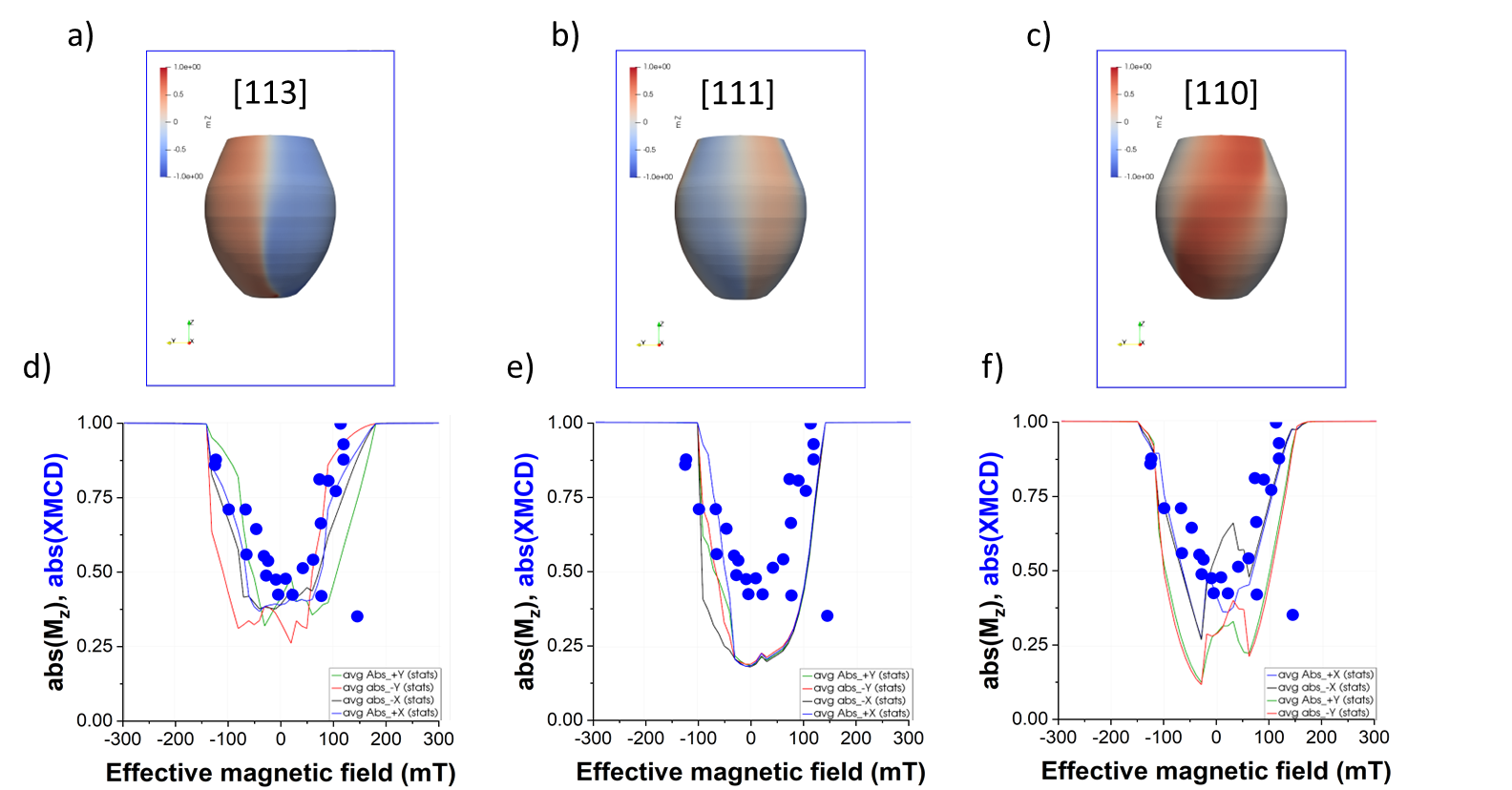


**Figure S9** a)-c) Micromagnetic simulations of the z-component of the magnetization for the fossil at 0 mT (after magnetic saturation) considering a [113], [111], and [110] crystallographic orientation along the long axis, respectively. d)-e) comparison of the spatially resolved integration of the absolute value of the XMCD (blue dots) with the spatially resolved integration of the z-component of the magnetization obtained from micromagnetic simulations. As in the experiment, the tip of the fossil has not been included, and the integration has been restricted to a near-surface region of 5 nm to make it comparable to the surface-sensitive XMCD-PEEM experiments.

**3.- Three-dimensional vortex structure.**

Micromagnetic simulations show a 3D magnetic vortex ground state for the giant spearhead magnetofossil (Figure S10), in agreement with Harrison et al. (Ref. ^2^). The vortex core increasingly bends as the easy axis tilts away from its long axis.


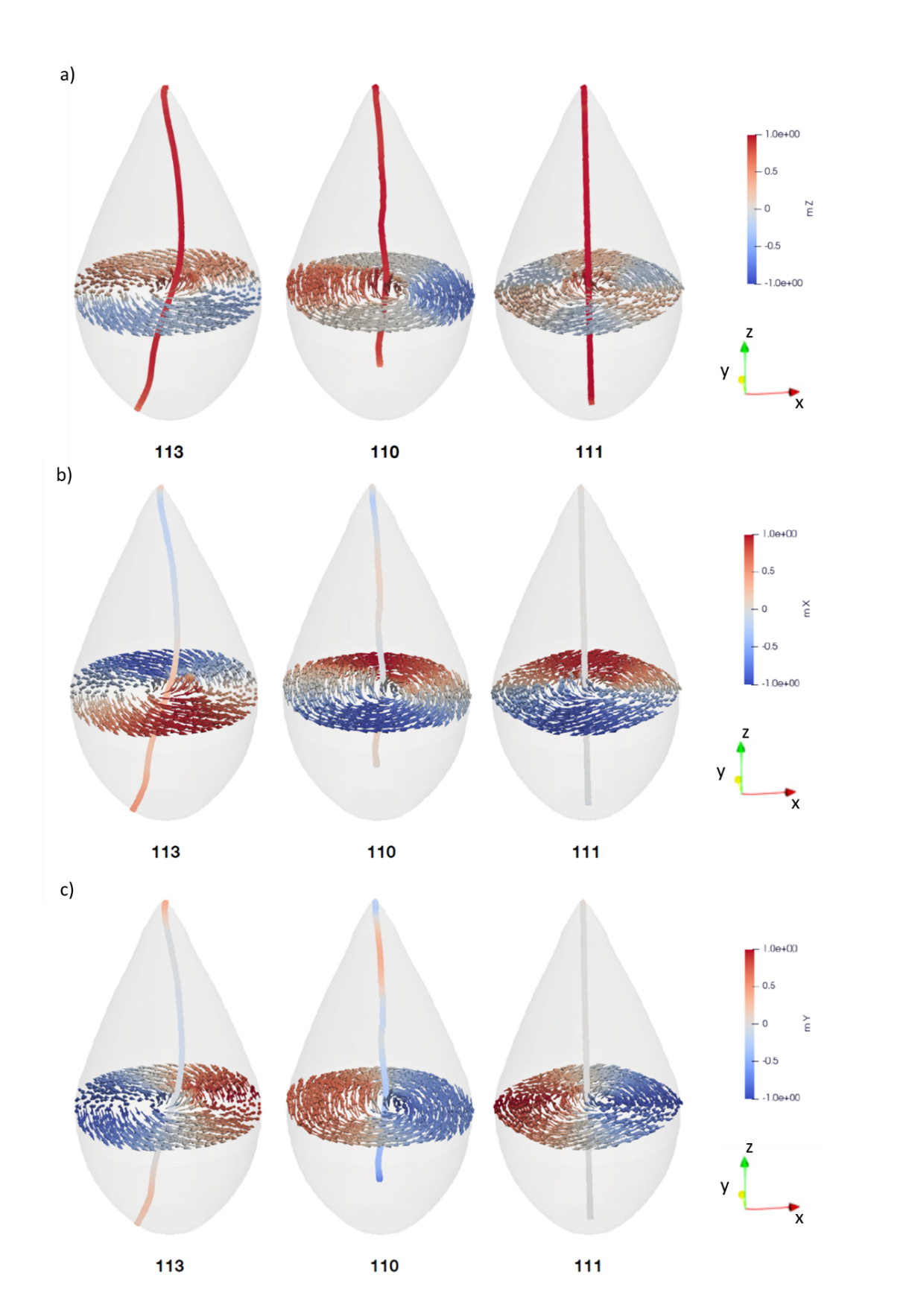


**Figure S10** Micromagneitc simulations with the [113], [110] or [111] cubic crystallographic directions aligned along the long axis of the particle. Two-dimensional contour projection of the giant magnetofossil (gray) including the vortex core and a XY section which highlights the vortex structure. Arrows indicate the local magnetization direction. a) z-component of magnetization, b) x-component of magnetization, and c) y-component of magnetization.

**4.- Geometrical and material parameters influence of MFC performance**

Figure 6 within the main text depicts the influence of some geometric parameters on the maximum magnetic field contribution of the concentrator, and on its magnetization reversal mechanism. The main text discusses the influence of the thickness *t*, the gap width *d_gap_*, and the number of petals, as well as the choice of material. However, other geometrical parameters can influence the magnetic behavior of the concentrator and need to be considered for an optimized design of the MFC taking into account both, sample and experimental constraints.

- An increase of the length of the petals, $L=R_{o}-R_{i}$, gives rise to a slightly higher concentrated field (Figure S11a). This enhancement of concentration field becomes less important as the petals are long. Below a threshold ($R_{O}\approx3R_{i}$) a strong reduction of the coercive field and an increase of the saturation field are observed, as the device shows the magnetic behavior similar to that of a plain disc.
- An increase of the radius of the disc, $R_{i}$, helps to reduce the coercive field, the maximum field generated by the MFC staying unchanged (Figure S11b). This can be explained as the additional ferromagnetic material brought by increasing R_i_ is relatively far for the center compared to the rest of the disc. Therefore, this added material contributes very little to the field generated at the center, but contributes to the demagnetizing energy of the disc, favoring the apparition of domains and therefore lowering the coercive field.
- An alteration of the petal arrangement changes the reversal magnetization dynamics while keeping the maximum field generated by the MFC unchanged (Figure S11c). The missing petals fail to pin the magnetization direction at the circumference of the disc, leading to an easier formation of domains in the disc during the magnetization reversal.

In addition to the geometry of the MFC, the presence of a ferromagnetic sample in the gap is susceptible to altering the magnetic behavior of the MFC. This effect has been tested by computing the magnetic hysteresis loop of a standard MFC including magnetic particles of different sizes inside the gap. As shown in Figure S11d, even the largest considered sample —- barely touching the MFC edge (a single discretization cell is separating the sample from the MFC) — have negligible influence on the magnetization of the MFC as does not perturb its core dynamics.

_
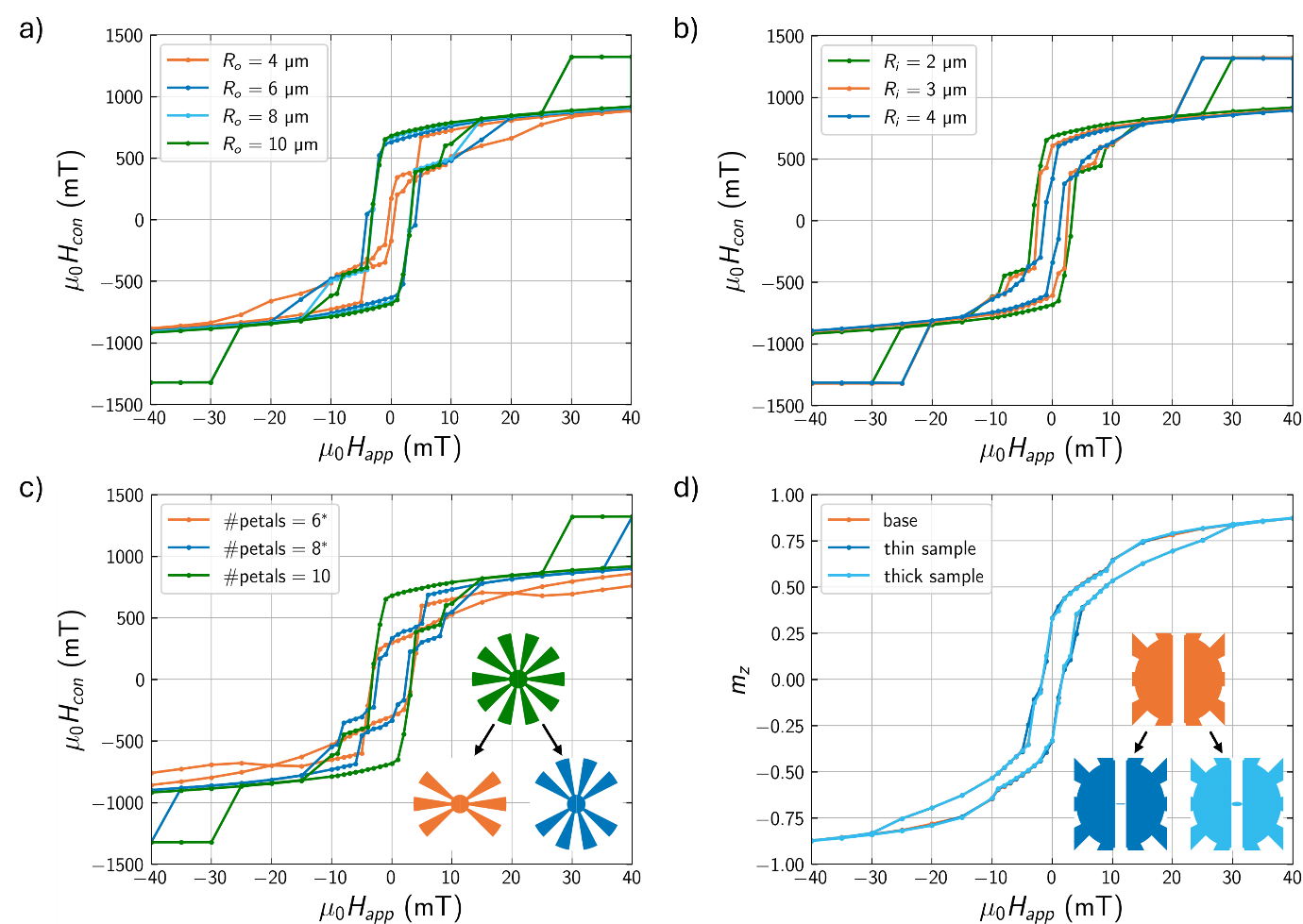
_

**Figure S11** a-c) Micromagnetic simulations of the field created by cobalt MFCs (H_con_) at its gap center as function of applied field (H_app_) for different geometric and material parameters, varying; a) outer radius, b) internal radius, and c) material. Other parameters are fixed unless varied: $R_{O} = 5R_{I} = 10$, $t = 70$ nm and $d_{gap}$ = 50 nm. d) z-component of the normalized magnetization —along the applied field direction, i.e. across the gap— averaged over the MFC volume. Results are shown for the MFC alone and with a ferromagnetic magnetite ellipsoid particle in its gap: thin sample is 460 nm X 50 nm X 50 nm, and thick sample is 480 nm X 200 nm X 50 nm. MFC dimensions R_O_ = 5R_i_ = 10 μm, t = 70 nm and d_gap_ = 500 nm. Field applied across the gap.

**5.- Effect of enhanced and localized magnetic fields on Spatial cross talk and spatial resolution in PEEM**

Figure S12 compares the expected evolution of the in-plane magnetic field component above the surface computed for i) the gap of the sample holder [Kronast *et al.* *Surf. Interface Anal.* **42**, 1532-1536 (2010)] and ii) the center of an MFC with dimensions alike to those used for the study of the magnetosome chain. The magnetic field created by the sample holder decays to about 20% of its original value after ca. 3 mm. In contrast, for the MFC used for the the magnetosome chain and the fossil, a similar decay occurs below 1 μm, see Figure S12. This strong confinement suggests that their impact in resolution and spatial cross talk is minimal.


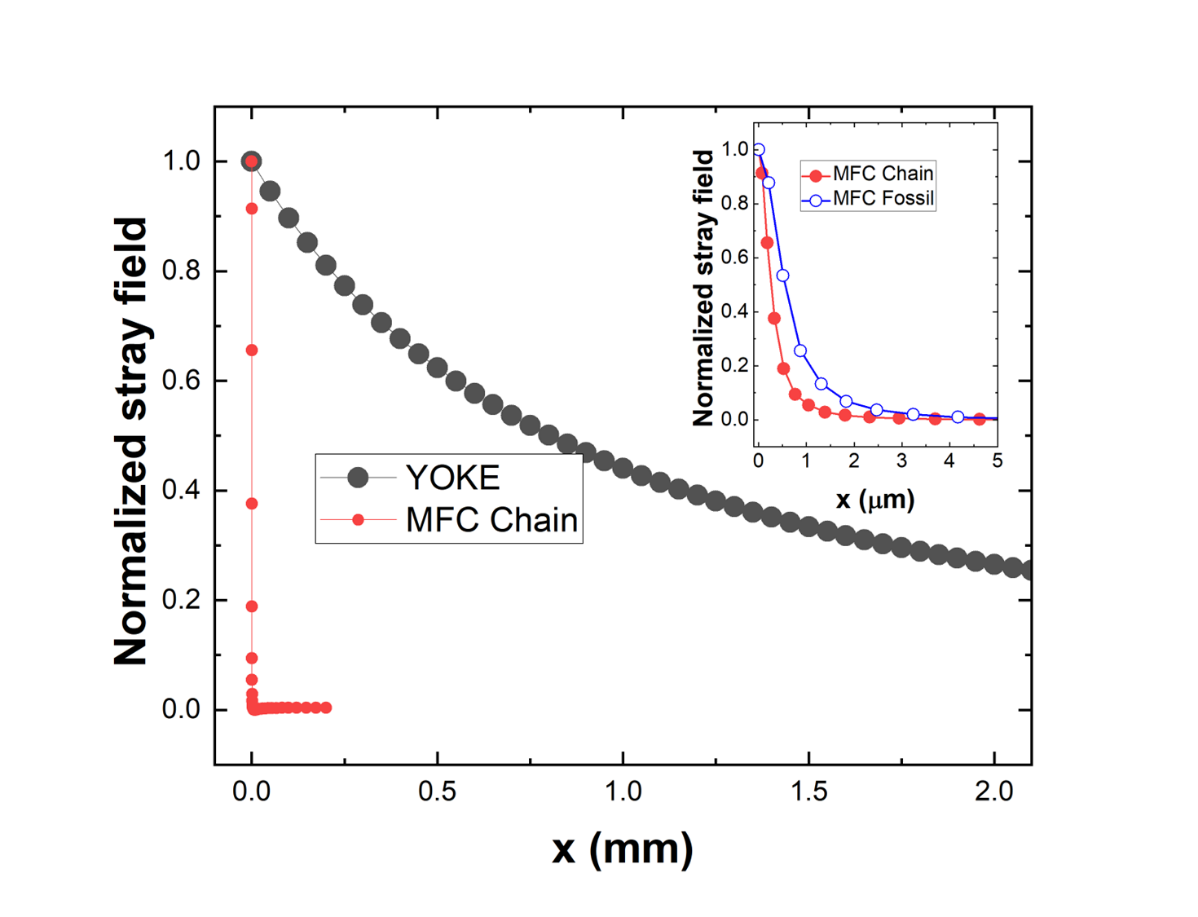


**Figure S12** Black curve) normalized field profile above the yoke surface (x) for the yoke implemented in the PEEM sample holder (Kronast et al. Surf. Interface Anal. **42**, 1532-1536 (2010)). Red curve) Normalized field profile above the surface of the MFC for magnetosome chain. Insect: zoom on first 5 micrometers above surface for the MFC showing surface field confinement by the MFC for magnetosome chain (red) and fossil (blue).

**Spatial cross talk**

Regarding a potential spatial cross talk in PEEM; in-plane magnetic fields deflect electrons originating from the sample. All electrons experience the same deflection if the field is homogeneous. On the contrary, an inhomogeneous field (B+ ΔB_z_) leads to different deflections causing electron trajectories to cross in their way towards the detector. The resulting lateral displacement can be calculated using the Lorentz force equation.

An electron moving towards the detector (X-axis) at a velocity $v_{x}$ in the pressence of a field inhomogeneity ΔB_z_ experiences an additional lateral acceleration

$${\Delta a}_{y}(x)=\frac{ev_{x}\left( x \right)}{m_{e}}\Delta B_{z}(y)$$

with e and m_e_ being the charge and the mass of the electron. The electron velocity along x v_x_(x) depends on its initial energy (E_0_ ~ 1 eV) and the energy acquired in the PEEM field potential (E_z_);

$$v_{x}\left( x \right)=\sqrt{\frac{2}{m_{e}}\left( E_{0}+E_{z} \right)}$$

The extra lateral velocity $v_{y}$ of the electron at a position x above the surface of the MFC is then

$${\Delta v}_{y}\left( x \right)= \int_{0}^{t} \Delta a_{y}\left( x_{1} \right)dt=\frac{e}{m_{e}}\int_{0}^{x} \Delta B_{z}\left( x_{1} \right)dx_{1}$$

using dt=dx_i_/v_x_, and t=x_i_/v_x_

The total lateral displacement after the electron have travelled a distance x is:

$\Delta y= \int_{0}^{t} \Delta v_{y}\left( x_{2} \right)dt=\frac{e}{m_{e}}\int_{0}^{t} \left[ \int_{0}^{x_{2}} \Delta z\left( x_{1} \right)dx_{1} \right]dt=\frac{e}{m_{e}}0x\frac{1}{v_{x}\left( x \right)}0x2\Delta$𝐵𝑥$z1dz_{1}{dz}_{2}$

We consider that the x-dependence of the field inhomogeneity follows the same trend as the total magnetic field above the MFC:

$$\Delta B_{z}\left( x \right)={\Delta B}_{z,max}\left( A_{1}e^{-x/b_{1}} \right)$$

Since the field is confined within ca. 0.7 μm above the surface (90% decay) we calculate the total lateral displacement accumulated at this position. At x = 0.7 μm, the total energy accumulated by the electrons is small (~4.5 eV for an acceleration field of 5 MV/m). This allows to approximate v_x_(x) by an effective velocity v_eff_ corresponding to the velocity averaged from its initial and final value (x =0.7 μm). With this simplification the cross talk reduces to

$$\Delta y= \frac{e{\Delta B}_{z,max}}{m_{e}v_{eff}}\left( A_{1}b_{1}\left( x-b_{1}\left( 1-e^{-x/b_{1}} \right) \right) \right)$$

At x= 0.7 μm v_eff_ ~ 9.27∙10^5^ m/s. The fit of the MFC field above its surface yields:

A_1_=1.055, b_1_= 0.339 μm

Plugging these numbers into the previous equation we obtain:

$\Delta y \sim0.03 nm/mT$

Thus, a cross talk comparable to the optimum spatial resolution (30 nm) would require field inhomogeneities of ~ 1000 mT, which is much larger than the fields generated by the MFC.

For the MFC used for the study of the fossil, shows a slightly slower decay (90% decay at x = 1.6 μm ). With v_eff_ ~ 1.19∙10^6^ m/s and the parameters adjusting the field profile

A_1_=1.057, b_1_= 0.702 μm

The lateral cross talk is:

$\Delta y \sim0.11 nm/mT$

A cross talk comparable to resolution would be take place in magnetic field inhomogeneities of approximately 300 mT.

Given the experimental conditions, the spatial cross talk in both cases studied within the main manuscript can safely be ignored.

**Spatial Resolution**

The presence of a magnetic field, even when homogeneous, does affect resolution. As shown, electron trajectories bend due to the presence of the magnetic field. This “bending” is different for electrons being emitted at different angles even when originating from the same position, thus leading to different lateral displacements and affecting resolution. Considering a given angular spread of the electrons, resolution will depend -as for the spatial cross talk case- on the integral of the field along the electron path.

In the following we provide a qualitative discussion on the effect of the MFC on the resolution by comparing its performance when mounted on top of a sample holder with that of the sample holder only.

We consider 3 situations depending on the effective field Heff, the applied field Happ, and the maximum field the yoke can provide during imaging *H*_app_(max):

1. ***H*_eff_ < *H*_app_(max)**

Figure S13a compares the spatial extent of the stray field above the sample surface for two systems delivering the same in-plane magnetic field: i) a conventional yoke (black curve), and ii) a conventional yoke + MFC (red curve). The system with the MFC requires much less applied fields to achieve same *H*_eff_. The strong confinement of the field near the sample surface provided by the MFC reduces the total Lorentz force and, thereby improves the resolution.


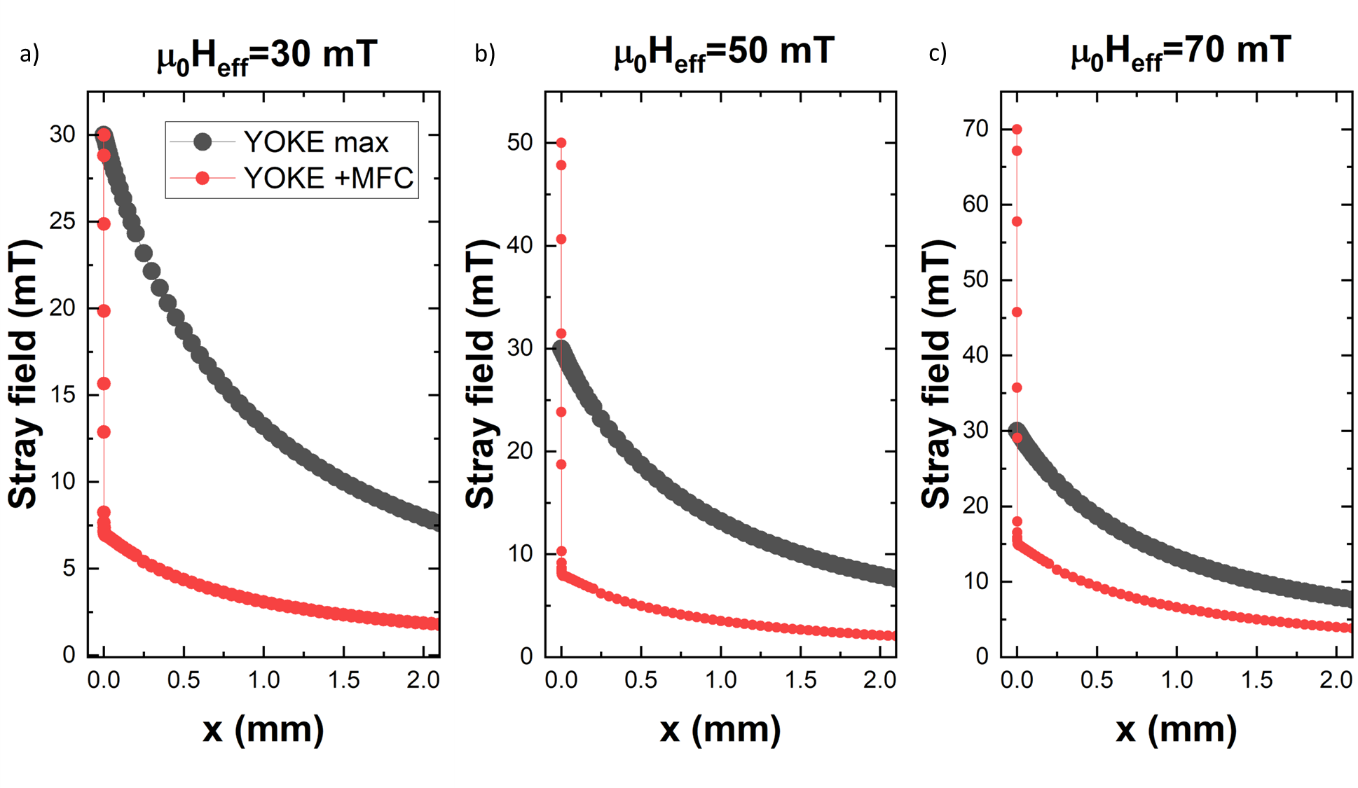


**Figure S13** Field profiles above sample surface (x) for the case of a yoke only (black) and for a yoke plus MFC (red). A) Effective field of 30 mT in both cases. For the MFC the applied field is 7 mT. b) Effective field of 50 mT for MFC with 8mT applied. C) effective field of 70 mT with 15 mT applied. Applied and effective field values extracted from Figure S3a for the case of the MFC associated to the magnetosome chain

1. ***H*_eff_ > *H*_app_ (max) > *H*_app_**

Given the strong localization of the magnetic field created by the MFC (a 90% decay within 0.7 μm), the integral of the magnetic field profile is expected to remain smaller than that of conventional yoke at its maximum applied field (Figure S13b). Consequently, even when the effective field is larger than possible using the conventional yoke sample holder, the resolution is expected to be better.

1. ***H*_eff_ > *H*_app_ (max)**

The magnetic field integral exceeds that of the conventional yoke operating at maximum applied field (Figure S13c). Despite the high localization of the field created by the MFC, a worsening of the resolution is expected to occur, its strength depending on *H*_eff_. Nevertheless, this degradation is expected to be small due to the spatial confinement of the field close to the sample surface.

**6.- Supplementary movies**

We include Supplementary movies (1 to 12) showing the field-dependent magnetic domain evolution of the fossil particle as seen along ± x and ± y directions for simulated [113], [111], and [100] crystallographic orientations along the long axis of the fossil.

- p1_113_+x.mp4
- p1_113_-x.mp4
- p1_113_+y.mp4
- p1_113_-y.mp4
- p1_110_+x.mp4
- p1_110_-x.mp4
- p1_110_+y.mp4
- p1_110_-y.mp4
- p1_111_+x.mp4
- p1_111_-x.mp4
- p1_111_+y.mp4
- p1_111_-y.mp4

**References**

1 Jamet, S. *et al.* Quantitative analysis of shadow x-ray magnetic circular dichroism photoemission electron microscopy. *Physical Review B* **92**, 144428 (2015). <https://doi.org:10.1103/PhysRevB.92.144428>

2 Harrison R.J. *et al.* Magnetic vector tomography reveals giant magnetofossils are optimised for magnetointensity reception. *Communications Earth & Environment*, in-press (2025).
